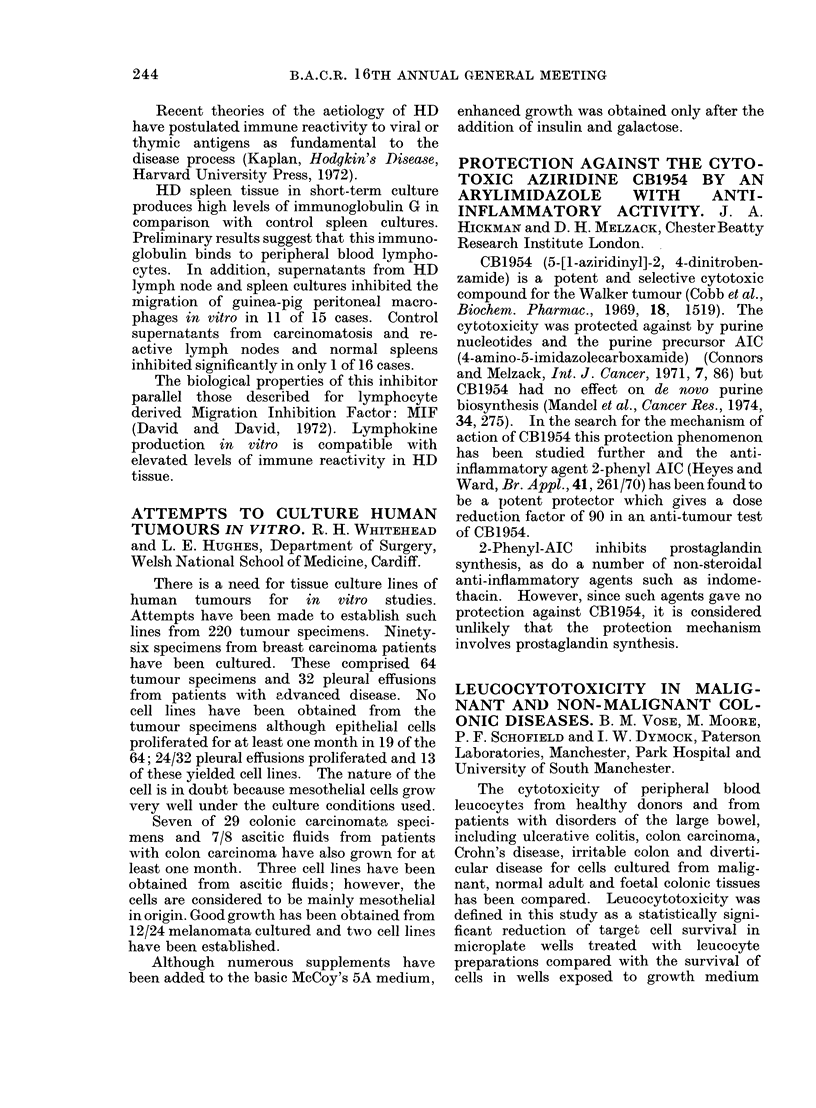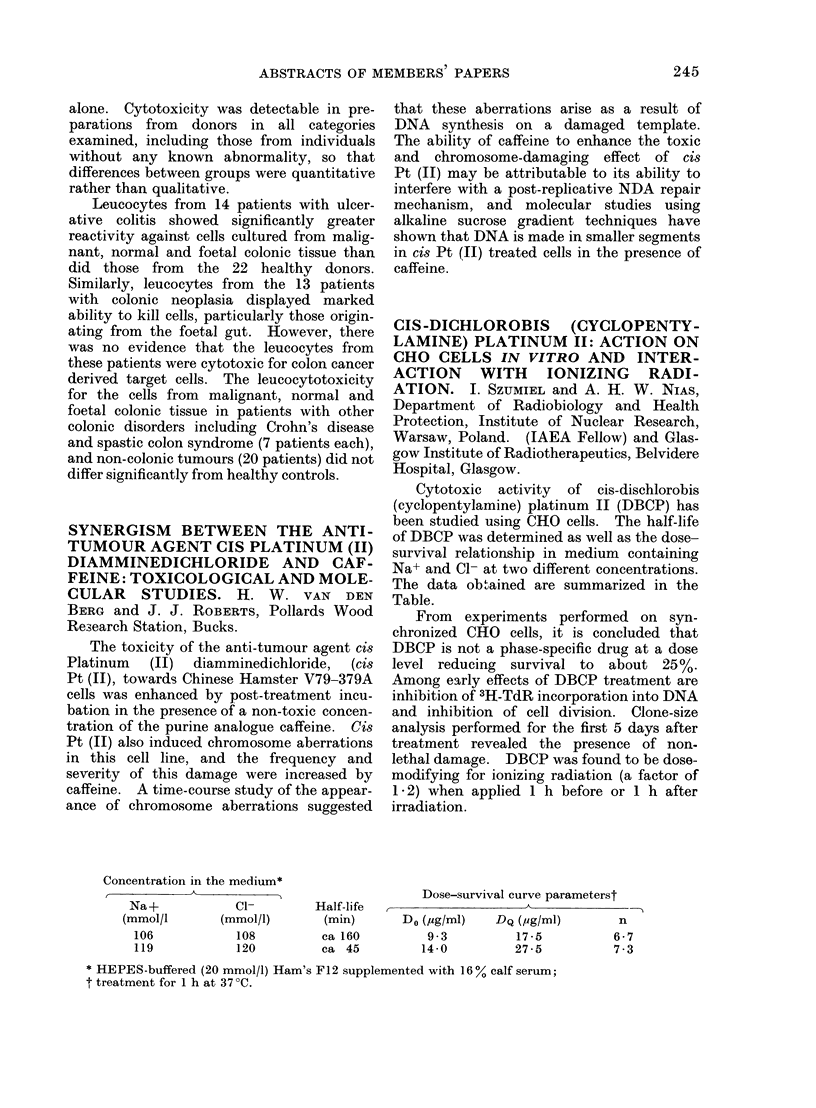# Proceedings: Leucocytotoxicity in malignant and non-malignant colonic diseases.

**DOI:** 10.1038/bjc.1975.170

**Published:** 1975-08

**Authors:** B. M. Vose, M. Moore, P. F. Schofield, I. W. Dymock


**LEUCOCYTOTOXICITY IN MALIGNANT AND NON-MALIGNANT COLONIC DISEASES.** B. M. VOSE, M. MOORE, P. F. SCHOFIELD and I. W. DYMOCK, Paterson Laboratories, Manchester, Park Hospital and University of South Manchester.

The cytotoxicity of peripheral blood leucocytes from healthy donors and from patients with disorders of the large bowel, including ulcerative colitis, colon carcinoma, Crohn's disease, irritable colon and diverticular disease for cells cultured from malignant, normal adult and foetal colonic tissues has been compared. Leucocytotoxicity was defined in this study as a statistically significant reduction of target cell survival in microplate wells treated with leucocyte preparations compared with the survival of cells in wells exposed to growth medium alone. Cytotoxicity was detectable in preparations from donors in all categories examined, including those from individuals without any known abnormality, so that differences between groups were quantitative rather than qualitative.

Leucocytes from 14 patients with ulcerative colitis showed significantly greater reactivity against cells cultured from malignant, normal and foetal colonic tissue than did those from the 22 healthy donors. Similarly, leucocytes from the 13 patients with colonic neoplasia displayed marked ability to kill cells, particularly those originating from the foetal gut. However, there was no evidence that the leucocytes from these patients were cytotoxic for colon cancer derived target cells. The leucocytotoxicity for the cells from malignant, normal and foetal colonic tissue in patients with other colonic disorders including Crohn's disease and spastic colon syndrome (7 patients each), and non-colonic tumours (20 patients) did not differ significantly from healthy controls.